# Fifty‐Four Tablets Too Many: A Case Report of Amlodipine and Losartan Overdose

**DOI:** 10.1002/ccr3.70457

**Published:** 2025-04-22

**Authors:** Nishan Uprety, Bishal Bhandari, Vinayak Dhungana, Sulav Neupane, Sunischit Chandra Neupane, Sujan Tamang, Samiksha Acharya

**Affiliations:** ^1^ Nepalese Army Institute of Health Sciences Kathmandu Nepal; ^2^ BP Koirala Memorial Cancer Hospital Bharatpur Chitwan Nepal

**Keywords:** amlodipine, angiotensin receptor blocker, calcium channel blocker, hyperinsulinemia–euglycemia, losartan, overdose

## Abstract

Overdoses of widely used cardiovascular drugs calcium channel blockers and angiotensin receptor blockers cause severe hemodynamic instability. Timely management with hyperinsulinemia–euglycemia therapy, vasopressors, and calcium gluconate is critical. Enhanced clinical vigilance, prompt intervention, and long‐term care, including psychological evaluation, are essential to address underlying factors and prevent recurrence of overdoses.

## Graphical abstract

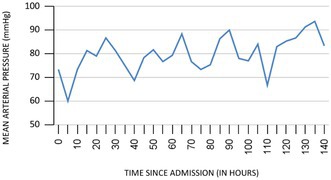

**Abbreviations**
ABG: arterial blood gas analysis
ALP: alkaline phosphatase
ALT: alanine aminotransaminase
ARB: angiotensin receptor blocker
CCB: calcium channel blockers
DNS: dextrose normal saline
ED: emergency department
GRBS: general random blood sugar
HIET: hyperinsulinemia–euglycemia therapy
ICU: intensive care unit
MAP: mean arterial pressure
NS: normal saline
TLC: total lymphocyte count

## 1 Introduction

### 1.1 Background

Calcium channel blockers (CCBs) are commonly prescribed medications to treat various medical conditions, including hypertension, supraventricular tachycardia, vasospasm, and migraine headaches [1]. The widespread use of CCBs has contributed to the increase in intentional and accidental overdoses that are linked to lethal consequences as a result of cardiovascular collapse [2]. The fixed combination of losartan and amlodipine has been shown to be safe and efficacious in the management of hypertension in patients [3, 4]. However, angiotensin receptor blocker (ARB) could produce synergistic toxicity in patients with an amlodipine overdose by limiting the effectiveness of both endogenous and exogenously administered catecholamines [5].

### 1.2 Rationale and Knowledge Gap

There have been a few reported cases of amlodipine and losartan overdose; however, literature is still sparse. The lack of standardized guidelines to manage amlodipine and losartan toxicity and the unfamiliarity of management among healthcare providers can lead to delays in management and poor outcomes in patients.

### 1.3 Objective

We present a case of a 47‐year‐old lady who presented to the emergency department (ED) after ingestion of a substantial amount of fixed‐dose pills of amlodipine and losartan.

## 2 Case History

A 47‐year‐old lady, weighing 61 kg, came to the ED accompanied by her husband. He gave a history of consumption of Mylod‐L (fixed dose combination of amlodipine and losartan) 4 h before arriving at the ED. She allegedly took 54 tablets of Mylod‐L, constituting 5 mg of amlodipine and 50 mg of losartan, equating to 270 mg (5 × 54) of amlodipine and 2700 mg (50 × 54) of losartan. She had a past medical history of hypertension and type 2 diabetes mellitus but no past psychiatric history. At presentation, she was well oriented to time, place, and person. She had a patent airway with blood pressure 100/60 mmHg, pulse rate 96 bpm, respiratory rate 22 bpm, temperature 98°F, and oxygen saturation 99% in room air.

### 2.1 Diagnosis

Amlodipine and losartan poisoning.

## 3 Methods

She was initially managed with a rapid infusion of a liter of IV fluid and 10 mL of 10% calcium gluconate. Constant monitoring was done. Her initial lab reports showed a normal total lymphocyte count, a deranged renal function test (creatinine 1.81 mg/dL, urea 54.5 mg/dL, sodium 130.8 mEq/L, and potassium 4.7 mEq/L), an increased blood sugar level of 416 mg/dL, a deranged liver function test (alanine aminotransferase 100.7 U/L, alkaline phosphatase 162 U/L, total bilirubin 0.63 mg/dL, and direct bilirubin 0.33 mg/dL), and a negative pregnancy test. Initial arterial blood gas analysis showed a blood pH of 7.31, a bicarbonate level of 20.32 mmol/L, and negative urinary ketones ruling out diabetic ketoacidosis.

**FIGURE 1:**
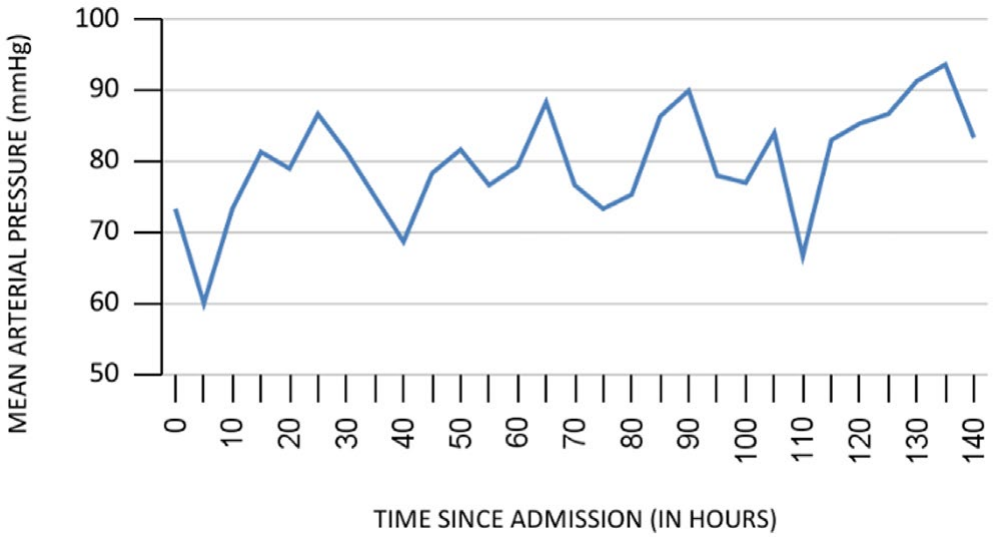
Chart showing the change in MAP over time.

The patient was shifted to the intensive care unit (ICU) due to persistently low blood pressure (80/60 mmHg) despite initial management at the ED (Figure 1). A central venous line and Foley catheter were placed. An infusion of noradrenaline was started at 0.3 mcg/kg/h following the administration of a liter of IV fluid. Calcium gluconate was continued hourly. Insulin infusion was started at 30 units/h along with normal saline (NS) at 100 mL/h due to her history of diabetes mellitus and initial blood glucose level. The target general random blood sugar was maintained between 160 and 200 mg/dL. When the blood glucose level dropped below 200 mg/dL, NS was changed to dextrose normal saline (DNS) or 5% DNS, as needed. She was also given pantoprazole (40 mg twice daily) and ondansetron (4 mg thrice daily) for symptomatic management of pain abdomen and nausea that the patient was complaining.

The management in the ICU steadily raised her mean arterial pressure (MAP) to more than 65 mmHg, which was the minimal target pressure. However, she remained anuric during the first day of admission.

**FIGURE 2:**
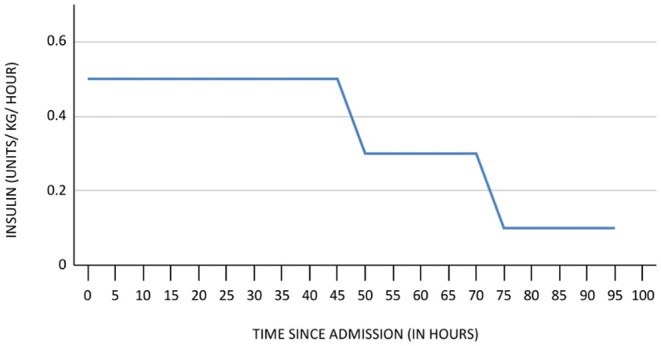
Chart showing the rate of insulin infusion over time.

Insulin (30 U/h at rate of 0.5 U/kg/h) and DNS (at rate of 100 mL/h) were continued along with calcium gluconate and infusion of noradrenaline at the same dose as before on the second day. Insulin was tapered down gradually and a basal bolus regimen was commenced during her stay in ICU (Figure 2). The infusion rate of noradrenaline was reduced from 0.3 to 0.1 to a further 0.06 mcg/kg/h over 3 days and subsequently discontinued to maintain the targeted MAP.

Ultrasonography of the abdomen and pelvis was unremarkable except for Grade I fatty liver. Urinary output was noted beginning on the second day of admission.

Oral diet was started on the third day with the continuation of the same treatment.

### 3.1 Outcome/Follow‐up

She was shifted to the high dependency unit after a stay of 5 days in ICU, where she was stable and asymptomatic. A psychiatric consultation evaluating her mental status, along with psychological counseling, was done. She was discharged from the hospital after a few days stay in the unit.

## 4 Discussion

Our case emphasizes the use of hyperinsulinemia–euglycemia therapy for the management of severe CCB and angiotensin II receptor blocker overdose. Most of the previously published case reports have mentioned using either metaraminol and/or hyperinsulinemia–euglycemia therapy (HIET) to maintain the MAP [6]. The paper by Zaher et al. has proposed that HIET appears to provide more synchronized myocardial contraction than other conventional therapies [7]. Though a few studies have reported the failure of HIET, it might be due to the fact that it was used late in the treatment as a rescue measure. In contrast, our case demonstrates that early use of HIET along with vasopressors can successfully maintain the target MAP.

Amlodipine, a dihydropyridine CCB, is widely utilized for the management of hypertension and angina due to its significant vasoselective properties that facilitate vascular smooth muscle relaxation [1]. By inhibiting L‐type calcium channels, amlodipine reduces intracellular calcium levels, leading to diminished vascular resistance. Losartan, an ARB, acts by selectively blocking the AT1 receptor, inhibiting the vasoconstrictive and aldosterone‐secreting effects of angiotensin II [8]. In combination, these medications provide synergistic antihypertensive effects but, in overdose situations, can compound the suppression of vascular tone and exacerbate hypotension [2].

The patient presented to the ED with stable vital signs despite the ingestion of a substantial dose of Mylod‐L (270 mg of amlodipine and 2700 mg of losartan). Initial management with rapid IV fluid infusion was started. After blood pressure started to drop in a few hours during the stay at ED, HIET, along with NS/DNS/5% DNS, was started, aimed at counteracting calcium channel blockade and maintaining hemodynamic stability. Insulin therapy appears to shift the cellular metabolism from fatty acids to carbohydrates required in stress conditions in the myocardium and vascular smooth muscles, resulting in improved cardiac contractility and decreased peripheral resistances [7]. However, persistent hypotension (80/50 mmHg) necessitated escalation to intensive care and commencement of vasopressors.

In the ICU, a central venous line was placed, and noradrenaline infusion was started at 0.3 mcg/kg/h to maintain MAP. Literature supports the use of vasopressors in cases of CCB and ARB overdose when fluid resuscitation and calcium administration alone are insufficient [6, 9]. High‐dose insulin euglycemia therapy (HIET), initiated at 0.5 units/kg/h with concurrent dextrose infusion, played a pivotal role in stabilizing the patient's hemodynamics. HIET's positive inotropic effects, facilitated by enhanced myocardial glucose uptake and intracellular calcium levels, are well‐documented in severe CCB overdose management [1, 5].

The continued administration of insulin, calcium gluconate, and titration of noradrenaline throughout the second day resulted in sustained MAP and the eventual return of urinary output since the second day of ICU admission. These outcomes are consistent with documented cases that demonstrate the effectiveness of combined pharmacologic interventions in achieving hemodynamic stability [3, 10].

While in our case, the patient improved significantly with HIET and vasopressor, it alone cannot provide definitive effectiveness of HIET. The limited reported case reports of amlodipine and losartan poisoning and even fewer demonstrating the effectiveness of HIET in improving cardiac contractility and maintaining MAP necessitate further clinical studies to confirm the efficacy of HIET. We hope that this case report hopefully provides some valuable information and serves as a stepping stone for further future studies to validate the use of HIET for the management of amlodipine and losartan poisoning.

## 5 Conclusion

This case highlights the importance of a comprehensive, time‐efficient approach in managing severe CCB and ARB overdoses, demonstrating the effective use of HIET, vasopressors, and calcium gluconate as critical early interventions. Even though the case aligns with the existing treatment protocols, it still emphasizes the need for enhanced clinical vigilance and prompt, aggressive management and long‐term care, including psychological evaluation, to address underlying factors and prevent recurrence. Further research, such as multicenter clinical studies, is essential to validate and refine these strategies, while structured follow‐up care is crucial for optimizing patient outcomes.

## Author Contributions

**Nishan Uprety:** conceptualization, data curation, investigation, project administration, supervision, visualization, writing – review and editing. **Bishal Bhandari:** conceptualization, data curation, investigation, methodology, supervision, writing – review and editing. **Vinayak Dhungana:** formal analysis, methodology, project administration, resources, visualization, writing – original draft, writing – review and editing. **Sulav Neupane:** conceptualization, investigation, methodology, project administration, resources, visualization, writing – original draft, writing – review and editing. **Sunischit Chandra Neupane:** conceptualization, data curation, formal analysis, project administration, writing – original draft, writing – review and editing. **Sujan Tamang:** data curation, investigation, project administration, resources, writing – original draft, writing – review and editing. **Samiksha Acharya:** conceptualization, investigation, supervision, writing – review and editing.

## Ethics Statement

The authors are accountable for all aspects of the work in ensuring that questions related to the accuracy or integrity of any part of the work are appropriately investigated and resolved. All procedures performed in this study were in accordance with the ethical standards of the institutional and national research committee(s) and with the Helsinki Declaration (as revised in 2013).

## Consent

Written informed consent was obtained from the patient for publication of this case report and accompanying images. A copy of the written consent is available for review by the Editor‐in‐Chief of this journal on request.

## Conflicts of Interest

The authors declare no conflicts of interest.

## Data Availability

Data sharing not applicable to this article as no datasets were generated or analyzed during the current study.
